# Plasma C-C Chemokine Concentrations in Intermediate Age-Related Macular Degeneration

**DOI:** 10.3389/fmed.2021.710595

**Published:** 2021-11-18

**Authors:** Alan G. Palestine, Brandie D. Wagner, Jennifer L. Patnaik, Rebecca Baldermann, Marc T. Mathias, Naresh Mandava, Anne M. Lynch

**Affiliations:** ^1^Department of Ophthalmology, University of Colorado School of Medicine, Aurora, CO, United States; ^2^Department of Biostatistics and Informatics, University of Colorado School of Public Health, Aurora, CO, United States; ^3^Colorado Clinical and Translational Sciences Institute, University of Colorado, Anschutz Medical Campus, Aurora, CO, United States

**Keywords:** age-related macular degeneration, inflammation, chemokines, CCL2, CCL3, CCL4, CCL5, RANTES

## Abstract

**Purpose:** To determine the relationship between plasma concentrations of the C-C chemokines CCL2, CCL3, CCL4, and CCL5 and intermediate age-related macular degeneration (iAMD) patients compared with control inidividuals to further define the inflammatory pathways associated with age-related macular degeneration.

**Methods:** The concentrations of CCL2, CCL3, CCL4, and CCL5 were measured using multiplex assays in plasma collected from 210 patients with iAMD and 102 control individuals with no macular degeneration as defined by multi-modal imaging. Non-inflammatory data included in the analysis were: age, sex, family history of AMD, history of smoking, body mass index, presence of reticular pseudo-drusen and cardiovascular disease. Median concentrations as well as a cutoff value for each chemokine were compared between the two groups.

**Results:** The median concentrations of CCL2 and CCL4 did not differ between control and iAMD groups, however, CCL2 was elevated in iAMD when a cutoff comparison was used (*p* < 0.05). Median CCL3 and CCL5 concentrations were significantly decreased in the macular degeneration group compared with controls (*p* < 0.001) as well as when a cutoff value comparison was used. CCL3 and CCL5 were negatively correlated in cases and positively correlated in controls.

**Conclusions:** Plasma CCL3 and CCL5 concentrations were significantly decreased and CCL2 concentrations were increased in patients with iAMD compared with controls, suggesting a role for C-C chemokines in the systemic inflammatory processes associated with disease development.

## Introduction

Inflammation mediated by the innate immune system has a prominent role in the pathophysiology of age-related macular degeneration (AMD) and has been identified as a driving and potentially modifiable factor in the progression of this ocular disease (1). AMD is a progressive degenerative disease of the aging retina which accounts for significant visual loss in older patients. The early (eAMD) and intermediate (iAMD) stages of AMD are characterized by the presence of drusen and the development of pigmentary alterations in the retinal pigment epithelium (RPE) whereas advanced AMD can be divided into neovascular AMD (NVAMD) and geographic atrophy (GA) (2). It is the advanced forms of this disease that lead to visual loss, but intervention while the patient has iAMD has the potential to prevent future visual damage. Furthermore, the risk of developing AMD is associated with a number of systemic conditions including cardiovascular disease, obesity and smoking and these conditions are associated with systemic inflammatory markers (3). It is therefore important to determine how inflammation affects the risk of iAMD development and which inflammatory pathways may be involved.

Much of the emphasis of the role of inflammation in AMD has been directed toward the complement system, which is a pathway of soluble mediators involved in both cellular damage and chemotaxis. Specifically, mutations in complement factor H (CFH) have been shown to increase the risk of both AMD development and progression by increasing activation of the alternative pathway of complement (4). The most common variant in CFH is the Y402H mutation, however alterations in plasma complement levels in AMD are not correlated with the Y402H CFH mutation (5). The primary mediator of damage by complement activation has focused on the generation of C3b and the eventual production of the C5b-9 membrane attack complex (MAC) which can directly damage cells. Less emphasis has been placed on studying the cellular components of the innate immune system and the cytokines which regulate these cells, even though C3a and C5a are potent chemotactic factors which are increased during complement activation. We have previously shown that C3a and C5a are elevated in the plasma of patients with iAMD compared to controls (6) and hence chemotaxis of the cellular components of the innate immune system may also be important in AMD pathogenesis.

The C-C chemokines are additional soluble protein mediators of chemotaxis for the innate immune system. C-C chemokines (CCLx) are 8–10 kD proteins characterized by two cysteine di-sulfide bonds and attract a variety of inflammatory cells depending on the specific chemokine and the chemokine receptors expressed on a specific cell including monocytes, NK cells, eosinophils and lymphocytes (7). CCL2 has been shown to be of possible importance in an experimental model of AMD where CCL2 deficient mice develop features of AMD including drusen-like structures at the level of the RPE, RPE disruption and photoreceptor degeneration (8). These mice show recruited macrophages and microglia and it is hypothesized that these macrophages in the absence of CCL2 cannot phagocytize C5a, leading to retinal degeneration (9). In addition, the RPE of these CCL2 deficient mice have decreased ability to remove waste products in the sub-retinal space and there is increased expression of CCL5 in CCL2 deficient mice (9). Furthermore, patients who have single nucleotide polymorphisms in CCL2 and its receptor CCR2 are at higher risk of developing advanced AMD (10). Hence, C-C chemokines may play a significant role in the development of AMD and specifically be important in development of drusen and RPE abnormalities. We studied plasma concentrations of CCL2, CCL3, CCL4, and CCL5 [also referred to as macrophage chemo-attractant protein-1 (MCP-1), macrophage inflammatory protein-1 alpha (MIP1α), macrophage inflammatory protein-1 beta (MIPβ), and RANTES, respectively] in patients with iAMD to improve our understanding of the systemic inflammatory milieu that exists in patients who are at-risk to develop AMD. We hypothesized that C-C chemokine concentrations would differ in patients with iAMD compared to controls.

## Methods

We conducted this study on patients with iAMD who were recruited into the Colorado AMD registry (described in detail elsewhere) (6, 11). This registry is approved by the Colorado Multiple Institutional Review Board, utilizes a signed consent form and conforms to the Declarations of Helsinki. Patients included in the registry are recruited from the retina clinics at the UCHealth Sue Anschutz-Rodgers Eye Center. Informed consent is obtained from subjects after explanation of the nature and possible consequences of the study.

The recruitment, exclusion/inclusion criteria, and informed consent of each participant are described in detail elsewhere (6, 11). Briefly, patients are consented for: medical history review including family history, collection of serum and plasma samples for future biomarker studies, and review of multi-modal image data including fundus photography fundus autofluorescence and spectral domain ophthalmic coherence tomography (6, 11). Ocular exclusion criteria for the registry include: pan-retinal photocoagulation or anti-VEGF injections for diabetic retinopathy, branch, and central retinal vein occlusion (with severe macular damage), any active ocular inflammatory disease, or a severe decrease in visual acuity secondary to a preexisting severe retinal disease other than AMD. Control patients are cataract surgery patients over the age of 70 enrolled 1 month after cataract surgery who are confirmed to have no evidence of AMD by review of multimodal imaging.

### Image Review and Case Selection

The multimodal imaging described above is reviewed by two vitreo-retinal specialists, focusing on an examination of the anatomic macula, including the entire area between the retinal vascular arcades. All images are categorized into early, intermediate (iAMD), and advanced AMD (6, 11) using the classification described by Ferris et al. (12). Discrepancies are resolved by a third vitreo-retinal specialist. The iAMD cohort used for this study is a sub-cohort from the Colorado AMD registry. Intermediate AMD was defined as large drusen (>125 um) or pigmentary abnormalities associated with at least medium drusen (12) in either eye with no evidence of advanced AMD in either eye as assessed by fundus photography, spectral domain ophthalmic computerized tomography and fundus autofluorescence. Non-inflammatory risk factors included in the analysis were: age, sex, reported family history of AMD, body mass index, presence of reticular pseudo-drusen (RPD) on optical coherence tomography and select vascular co-morbidities.

#### Collection and Processing of the Blood Sample

Following phlebotomy, the Ethylenediaminetetraacetic acid tube was spun at 3,000 revolutions per minute (rpm) in a cooled (4° Celsius) centrifuge for 10 min to isolate plasma. The average time from phlebotomy to spin was low at 2.6 min ± 1.7 SD, range 0–10 min. All samples were pipetted into aliquots and stored at −80°C.

### Measurement of Plasma CC Chemokines

CCL2 (MCP-1), CCL3 (MIP1α), CCL4 (MIPβ), and CCL5 (RANTES) were measured at the Clinical Translational Core (CTRC) laboratory, located at Childrens' Hospital Colorado. Multiplex assays were performed using multiplex kits manufactured by R&D Systems that utilize color-coded microparticles coated with analyte-specific antibodies that are analyzed on dual-laser suspension array platforms. Specifically 150 microliters of plasma were analyzed using a magnetic bead-based multiplex method and read on the Luminex® FlexMap platform. All samples were performed in duplicate and had an acceptance threshold coefficient of variance of <15%.

### Statistical Analysis

Patient characteristics were compared between groups using either a chi-square test or Fisher's exact test, as appropriate, for categorical variables and a two-sample *t*-test for continuous variables. For CCL3, values below the lower limit of detection (LLOD) were randomly imputed using a uniform distribution between 0 and the LLOD. Analytes were log transformed (base 10 and anchored at 1). Chemokine values were compared between groups using a Wilcoxon ranked-sum test. Odds ratios (OR) and corresponding 95% confidence intervals (CI) were estimated using univariate logistic regressions. Cutoff values for the chemokines were determined using the Receiver Operator Curve (ROC) and the Youden index (13). Associations between chemokines were tested using Spearman rank-based correlation coefficients (Spr). All analyses were performed using SAS version 9.4 (The SAS Institute, Cary, NC).

## Results

Plasma samples were analyzed from 312 subjects in the registry, 210 with iAMD and 102 controls. Table 1 delineates the demographic characteristics of the iAMD and control cohorts. As expected, patients with iAMD were significantly more likely to have a family history of AMD than controls. The iAMD cases also had a higher incidence of stroke and were slightly older.

**Table 1:** Differences in clinical characteristics between subjects with intermediate AMD and controls.

	**Intermediate AMD** **(*n* = 210)**	**Controls** **(*n* = 102)**	**OR (95% CI)**	***P*-value**
Sex, female	127 (60%)	64 (63%)	0.9 (0.6–1.5)	0.70
**Family history of AMD**				
None	109 (52%)	81 (79%)	1	<0.001
Yes	72 (34%)	16 (16%)	3.3 (1.8–6.3)	
Uncertain	29 (14%)	5 (5%)	4.3 (1.7–13.1)	
Caucasian	202 (96%)	89 (87%)	3.7 (1.5–9.6)	0.003
Hispanic	4 (2%)	4 (4%)	0.5 (0.1–2.0)	0.45^†^
Age, Mean (SD)	77 (7.1)	75 (4.4)	1.6 (1.1–2.4)^*^	0.01
Body Mass Index, *n*	201	100	1.0 (0.9–1.1)	0.73
Mean (SD)	26.8 (5.2)	27.0 (5.4)		
**Smoking**				
Never	97 (46%)	52 (51%)	1	0.52^†^
Current	6 (3%)	1 (1%)	–	
Former	107 (51%)	49 (48%)	1.2 (0.7–1.9)	
**History of**				
Treated hypertension	112 (53%)	57 (56%)	0.9 (0.6–1.5)	0.67
Kidney disease	26 (12%)	12 (12%)	1.1 (0.5–2.3)	0.88
Stroke	13 (6%)	1 (1%)	–	0.04^†^
Peripheral vascular disease	33 (16%)	21 (21%)	0.7 (0.4–1.3)	0.29
Cardiac disease	74 (35%)	34 (33%)	1.0 (0.7–1.8)	0.74

*p-values obtained from Chi-square for categorical variables and t-test for continuous variables unless noted otherwise*.

* *OR corresponds to risk for every 10 year increase in age*.

† *p-value calculated from Fisher's exact test*.

**Figure 1:**
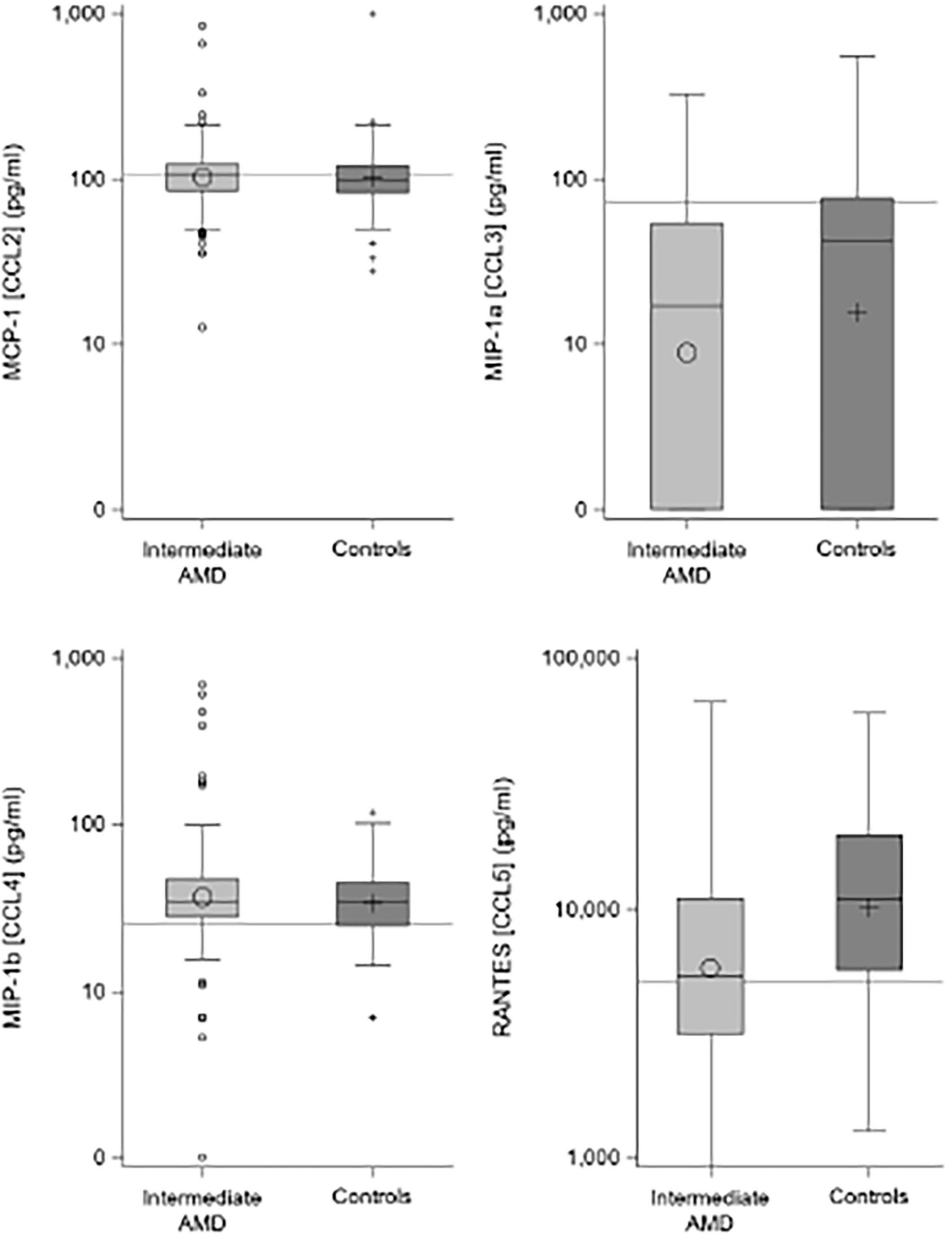
Boxplots comparing chemokine levels between iAMD and controls. Horizontal lines are the cutoff values from **Table 3**.

Table 2 shows the results of the plasma C-C chemokine analysis for the four measured proteins, demonstrating significantly lower median concentrations of CCL3 and CCL5 in iAMD cases compared to controls and Figure 1 illustrates this data in boxplot form. For CCL3, 39% of controls and 44% of iAMD patients were below the LLOD for this chemokine. CCL3 and CCL5 levels were both significantly lower in iAMD cases compared to controls (*p* < 0.001).

**Table 2:** Chemokine levels for cases with intermediate AMD vs. controls.

**Chemokines**	**Intermediate AMD (*n* = 210)**	**Controls** **(*n* = 102)**	***P*-value**
MCP-1 [CCL2] (pg/ml)	103.5 (84.6–123.2)	98.4 (82–120)	0.30
MCP-1a [CCL3] (pg/ml)	16.2 (0–52.8)	41.5 (0–76.2)	<0.001
MCP-1b [CCL4] (pg/ml)	34.6 (27.4–47.2)	34.3 (24.4–45)	0.45
RANTES [CCL5] (pg/ml)	5,374 (3,140–11,065.8)	11,028 (5,697–19,363)	<0.001

*Values displayed are the median (IQR), p-values obtained from Wilcoxon rank-sum test*.

**Figure 2:**
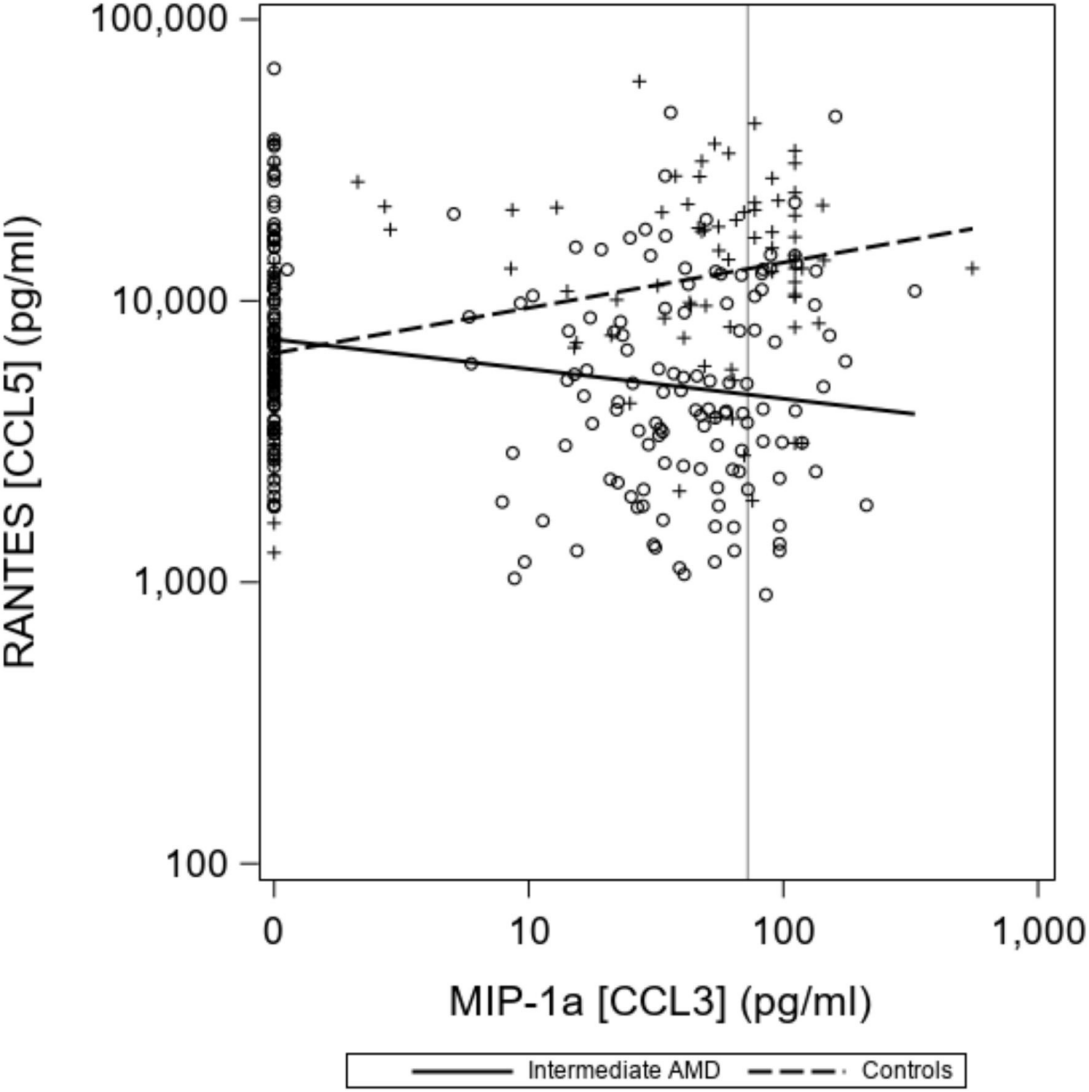
Correlations between chemokines CCL3 and CCL5 differ in controls and iAMD. The vertical line corresponds to the lower limit of detection (LLOD) for CCL3. Spearman Coefficient (95%CI) = −0.20 (−0.33, −0.07) in iAMD and = 0.37 (0.19, 0.53) for controls.

There were no differences seen in median CCL2 and CCL4 levels. Proposed optimal thresholds were selected for each chemokine using the Youden index (Table 3). There was again a significant difference between controls and iAMD in CCL3 and CCL5 levels (*p* < 0.001) using the cutoff values generated by the Youden index, but there was also a borderline significant difference in CCL2 between iAMD cases and controls (*p* = 0.05). The correlation between CCL3 and CCL5 was significant in both iAMD cases and controls, and the correlations differed between groups [Spr (95%CI) = −0.20 (−0.33, −0.07) in iAMD and Spr = 0.37 (0.19, 0.53)] (Figure 2).

**Table 3:** Chemokine cutoff levels from ROC curve analysis for discriminating between cases with intermediate AMD and controls.

**Chemokines**	**Cutoff level**	**Intermediate AMD** **(*n* = 210)**	**Controls** **(*n* = 102)**	***P*-value**
MCP-1 [CCL2] (pg/ml)	106	99 (47%)	36 (35%)	0.05
MCP-1a [CCL3] (pg/ml)	72^*^	30 (14%)	29 (28%)	<0.001
MCP-1b [CCL4] (pg/ml)	25	171 (81%)	74 (73%)	0.08
RANTES [CCL5] (pg/ml)	5,116	110 (52%)	83 (81%)	<0.001

* *Threshold for CCL3 is the LLOD, the optimal cutoff was not estimated*.

*N (%) of samples above cutoff level, p-values from Fisher's exact test using cutoffs*.

## Discussion

In the last two decades, it has become increasingly clear the inflammation as mediated by the innate immune system is important in the development of AMD. Although much of the understanding of this process has been focused on complement and specifically on CFH mutations, we believe that complement does not function in isolation in immune-mediated responses and that other soluble mediators as well as cellular components of inflammation may be important. For example, C reactive protein (CRP) has also been shown to be related to AMD development and we have shown that increased serum CRP is correlated with decreased choroidal thickness in iAMD (14). The final pathology in AMD is local damage to the RPE and outer retina, however the systemic inflammatory milieu may alter the local inflammatory responses. Obesity, smoking and cardiovascular disease are risk factors for AMD, suggesting that systemic factors can influence the local ocular environment.

Chemokines target and direct cellular migration of many of the cells of the innate immune system including macrophages, dendritic cells and natural killer cells, but also target lymphocytes of the adaptive immune system. There is evidence that alterations in C-C chemokines, and specifically CCL2, can produce many of the pathologic changes found in AMD in experimental animals (8, 9). CCL2 deficiency produces a pathology that resembles iAMD in mice, however our data did not show a strong relationship between median CCL2 concentrations and the risk for iAMD. The evidence suggesting that CCL2 deficiency produces retinal lesions that resemble AMD has been called into question because of the presence of the rd8 mutation in C57BL/6N strain as this mutation can also produce retinal degeneration (15). When we defined a cutoff value for CCL2, however, we were able to show a borderline relationship between CCL2 and the risk for iAMD in patients compared to controls. Further study of CCL2 is warranted based on the experimental model data as polymorphisms in CCL2 may be more important than absolute plasma concentration in defining the role of CCL2 as a risk factor for iAMD.

A much stronger relationship was seen for CCL3 and the risk of iAMD, but not for CCL4. These two chemokines are similar as they are also referred to as macrophage inflammatory protein alpha and beta, respectively. For example, CD4^+^ T cells and dendritic cells produce CCL3 and CCL4 when activated (16) and both chemokines recruit CD8+ cells (17). Both have been shown to increase with age (18) and are secreted together by monocytes and lymphocytes (19). However, in our study, there was a definite difference in the relationship of CCL3 and CCL4 to iAMD with no correlation seen for CCL4 and lower leverls of CCL3 seen in iAMD. Our study is limited, however, in that many patients with and without iAMD had plasma CCL3 levels below the LLOD in our assay. Further investigations with more sensitive assays may show a role for CCL3 in the evolution of AMD to advanced disease.

The most interesting chemokine in our study was CCL5, also known as RANTES (Regulated on Activation, Normal T cell Expressed and Secreted), which was significantly decreased in iAMD patients in our study. Decreased plasma levels of CCL5 have been associated with worse morbidity and mortality from cardiovascular disease (20), but elevated CCL5 has been associated with increased short term mortality in patients with acute coronary disease (21). Furthermore, elevated levels of CCL5 have been reported in patients with GA AMD compared to controls (22) and also in Parkinson's disease (23). Hence, alterations in CCL5 plasma concentrations may not simply be associated with aging but may have a role in regulating inflammatory processes in diseases of aging. Our finding that CCL5 is actually decreased in iAMD in contrast to the general notion that CCL5 increases with age suggests that this may be a marker of altered macrophage or T cell migration in patients with iAMD. Our findings are disparate from the recent report of elevated CCL5 in GA (22), suggesting that longitudinal studies could demonstrate changes in plasma CCL5 as AMD progresses. As noted above, CCL2 deficient mice have increased ocular expression of CCL5 (9). Hence, CCL5 may have a role both in iAMD development and in advanced AMD. There are several possible pathways to explore including the fact the CCL5 increased expression of multiple immune modulators including Il6 and TNF alpha in dendritic cells (24). The expression of the CCL5 receptor (CCR5) on CD8+ cells is negatively correlated with GA progression (22). The interaction of CCL5 with its receptor has been shown to have a role in neuroinflammatory diseases such as multiple sclerosis (25). Furthermore, increased secretion in human RPE cells of CCL5 (and CCL7) in response to cytokines such as TNF-alpha, IL1-beta, and IFN-gamma and the expression of CCR3 in choroidal blood vessels in patients with AMD suggests potential mechanism for both local and plasma CCL5 to interact with choroidal blood vessels and macrophage migration in AMD (26). Our finding of decreased plasma CCL3 and CCL5 in iAMD needs to be reconciled with the elevated levels of CCL5 reported in GA in order to speculate further on the mechansisms. Since CCL5 levels are not uniformly increased or decreased in aging diseases, the interaction of this chemokine and its receptor with ocular cells deserves further study.

The negative correlation between CCL3 and CCL5 in our iAMD patients as opposed to the positive correlation of these two chemokines in controls demonstrates a possible interaction between chemokine pathways. This has not previously been described and may point toward a unique feature of AMD pathogenesis and evolution. These correlations may represent differences in the systemic inflammatory milieu in AMD, suggesting the possibility that, while AMD is one of many degenerative aging diseases, it develops in a specific context of inflammation in the aging patient. It will be important to correlate the expression of chemokine receptors in autopsy specimens of patients with AMD to understand the significance of plasma chemokine alterations in AMD.

Limitations of our study include a cross sectional measurement of chemokine plasma levels at a single time point of enrollment in the registry in the presence of varying severity of iAMD pathology in the evolution of AMD in these patients. Longitudinal and repeated measurements of these chemokines in future studies will be of interest. Furthermore, our patients are recruited from a retina service and may not represent the spectrum of iAMD seen in the overall aging population.

The balance of up and down regulation of systemic chemokines, local chemokines, and chemokine receptors likely influences the development and progression of many neuro-degenerative diseases (27). It has been suggested that chemokine alterations prime the retina and choroid to develop degenerative aging changes (28), but it is also possible that plasma chemokines interact directly with choroidal blood vessels, altering macrophage migration into the eye. Our findings combined with observations of others suggest an important but not fully defined role of chemokines and their targeted control of inflammatory cellular traffic in the development of iAMD. The kinetics and longitudinal evolution of chemokine interactions with ocular tissue may prove to be useful in understanding the pathogenesis of AMD and provide new pathways for treatment and prevention.

## Data Availability Statement

The raw data supporting the conclusions of this article will be made available by the authors, without undue reservation.

## Ethics Statement

The studies involving human participants were reviewed and approved by Colorado Multiple Institutional Review Board, University of Colorado. The patients/participants provided their written informed consent to participate in this study.

## University of Colorado Retina Research Group

Rebecca Baldermann, Anne M. Lynch, Naresh Mandava, Marc T. Mathias, Scott C. N. Oliver, Jeffery L. Olson, Alan G. Palestine, Jennifer L. Patnaik, Paula E. Pecen, Frank S. Siringo, Jesse M. Smith, Brandie D. Wagner.

## Author Contributions

AP and AL: design, data acquisition, analysis, and manuscript writing. BW and RB: analysis and manuscript writing. JP: data acquisition, analysis, and manuscript writing. MM: data acquisition and manuscript writing. NM: design and manuscript writing. All authors contributed to the article and approved the submitted version.

## Funding

This research was supported by the National Eye Institute of the National Institutes of Health under award number R01EY032456 (AL), a Research to Prevent Blindness grant to the Department of Ophthalmology, University of Colorado, the Frederic C. Hamilton Macular Degeneration Center, Sue Anschutz-Rogers Eye Center Research Fund, and by NIH/NCATS Colorado CTSA Grant Number UL1 TR002535.

## Conflict of Interest

The authors declare that the research was conducted in the absence of any commercial or financial relationships that could be construed as a potential conflict of interest.

## Publisher's Note

All claims expressed in this article are solely those of the authors and do not necessarily represent those of their affiliated organizations, or those of the publisher, the editors and the reviewers. Any product that may be evaluated in this article, or claim that may be made by its manufacturer, is not guaranteed or endorsed by the publisher.
